# Leading health systems change through research from within West and Central African experiences

**DOI:** 10.4314/gmj.v56i3s.1

**Published:** 2022-09

**Authors:** Mary Amoakoh-Coleman, Emilie Pigeon-Gagne, Irene A Agyepong, Sue Godt

**Affiliations:** Noguchi Memorial Institute for Medical Research, College of Health Sciences, University of Ghana.0000; University of Montreal; Ghana College of Physicians and Surgeons, Public Health Faculty / Dodowa Health Research Center; Independent consultant

Health problems are often driven by complex embedded intertwined social determinants of health.^1^ Individual interventions, isolated from system considerations, rarely result in sustainable solutions. As noted by Rutter et al., “*Instead of asking whether an intervention works to fix a problem, researchers should aim to identify if and how it contributes to reshaping a system in favourable ways*”^2^ Research and capacity strengthening to generate and implement solutions need to be appropriate to the context and focus on policies and systems as well as specific interventions. There is a need to strengthen national and sub-national capacities and systems for contextually relevant evidence generation rather than just focusing on identifying “proven effective interventions” for transfer to varying contexts in a travelling models approach.^3^ This supplement presents experiences and research findings from efforts by West and Central African researchers to address pressing health problems collaboratively and to strengthen health policies and systems from within.

The West and Central African Partnership for Health Policy and Systems and Maternal Newborn Child and Adolescent Health, better known as the Consortium for Mothers, Children, Adolescents and Health Policy and Systems Strengthening (COMCAHPSS) started implementation in April 2016 after one year of sub-regional consultation to develop the program. COMCAHPSS has conducted multi-level capacity building, leadership strengthening and networking at the individual, institutional and country levels in West and Central Africa with funding support from International Development Research Centre (IDRC) Canada. Central to the effort has been South-South and anglophone-francophone collaboration, partnership, and networking by a consortium of academic, research, practice and civil society organisations and individuals, as well as collaboration with the West African Health Organization (WAHO).

The fourteen articles in this supplement can be categorised into three interrelated themes (1) strengthening an enabling health policy and systems research (HPSR) environment (2) generating evidence, and (3) using evidence to inform policy, program design and implementation. Priority and support have been given to encouraging papers and leadership by early and mid-career researchers from West and Central Africa, including the West African Network of Emerging Leaders (WANEL).

## Strengthening an enabling HPSR environment

Four papers focus on the processes, experiences and lessons needed to strengthen an enabling health policy and systems research environment through multi-level capacity building and networking. They cover the findings and lessons from the mid-term formative evaluation of COM-CAHPSS; the HIGHER Women program for mentoring young female health researchers in Cameroon; developing communities of practice in six countries, and historical and contemporary factors and processes that influenced the emergence of the West African Network of Emerging Leaders (WANEL).

## Generating evidence to inform policy, program design and implementation

Nine papers analyse and identify the status, challenges, and opportunities for strengthening health systems to improve programs and service delivery related to maternal, newborn, child and adolescent health and the response to Covid 19. Three of these papers are from Ghana, with two focused on MNCAH and one on COVID-19. Four studies are from Nigeria, with three focused on MNCAH and one on the health systems response to COVID-19. The paper from Nigeria by Kalu et al^4^ was published ahead of the other papers in this supplement. One multi-country paper led by an early career researcher from Niger analyses the supply and provision of family planning (FP) services to unmarried adolescents in Burkina Faso, Ghana, and Niger. The final paper in this cluster analyses the factors contributing to the evolution of morbidity and mortality in the ECOWAS during the COVID-19 pandemic.

## Using evidence to inform policy, program design and implementation

Johnson et al. present WANEL's knowledge translation framework of strategies and interventions and the required implementation capacities to address identified areas of intervention needed to influence health policies and practices in West Africa.
